# P-255. Receipt of Guideline Concordant Oncologic Care between Older HIV-Positive and Older HIV-Negative Patients with Cancer

**DOI:** 10.1093/ofid/ofaf695.476

**Published:** 2026-01-11

**Authors:** Uma Markan, David J Riedel, Habib Omari, Hannah Ashe, Jennie Y Law

**Affiliations:** University of Maryland Medical Center, Baltimore, MD; Institute of Human Virology, University of Maryland School of Medicine, Baltimore, MD; University of Maryland Baltimore, Baltimore, Maryland; University of Maryland School of Medicine, Baltimore, Maryland; University of Maryland Greenebaum Comprehensive Cancer Center UMGCCC, Baltimore, Maryland

## Abstract

**Background:**

With the success of antiretroviral therapy (ART), the population of people living with HIV (PLWH) is aging. The median age of PLWH on ART is projected to be 52 by 2030. PLWH experience worse cancer outcomes and are less likely to receive standard-of-care treatment compared to HIV-negative patients. We conducted a retrospective analysis to assess whether older PLWH (OPLWH, ≥50 years) receive guideline-concordant oncologic care compared to HIV-negative older patients (HNOP).

**Methods:**

A single institution retrospective review in which OPLWH and HNOP diagnosed with diffuse large B cell lymphoma (DLBCL), non-small cell lung cancer (NSCLC), hepatocellular carcinoma (HCC), and cholangiocarcinoma between the years of 2015-2022 were identified using our institutional cancer registry. Guideline concordance was defined as receipt of care aligned with NCCN guidelines from the year of cancer diagnosis. Chi square test was used to compare group characteristics. Multivariable logistic regression models were used to assess factors associated receipt of care. Adjusted odds ratio (aOR) and 95% CI were used to quantify the effect of these associations.

**Results:**

We evaluated 238 patients: 78 OPLWH and 160 HNOP. Median age was 61 (56–66) for OPLWH and 65 (59–71) for HNOP. 83% of OPLWH were Black, compared to 40% of HNOP. Guideline-concordant care was offered to 91% of OPLWH and 93% of HNOP (p=0.446). OPLWH had lower odds of receiving such care (84% vs 91%, p=0.128), though not statistically significant. Among those treated, 86.4% of OPLWH and 93.8% of HNOP tolerated therapy (p=0.08). Compared to publicly insured patients, uninsured patients were less likely to be offered (77.8% vs 92.8%, p=0.134), receive (66.7% vs 91.9%, p=0.06), and tolerate (66.7% vs 91.9%, p=0.06) treatment, though differences were not statistically significant.

**Conclusion:**

There was no significant difference in the provision of guideline-concordant care between OPLWH and HNOP with DLBCL, NSCLC, or hepatobiliary cancers. Both groups received and tolerated therapy similarly. To our knowledge, this is the first study examining guideline-concordant care in PLWH. These findings suggest progress in delivering evidence-based treatment to OPLWH, with lack of insurance emerging as a potentially greater barrier than HIV status alone.

**Disclosures:**

All Authors: No reported disclosures

